# The effect of individualized, theory-based counselling intervention on active aging and quality of life among older people (the AGNES intervention study)

**DOI:** 10.1007/s40520-020-01535-x

**Published:** 2020-04-01

**Authors:** Taina Rantanen, Mary Hassandra, Katja Pynnönen, Sini Siltanen, Katja Kokko, Laura Karavirta, Markku Kauppinen, Sarianna Sipilä, Milla Saajanaho, Erja Portegijs

**Affiliations:** Faculty of Sport and Health Sciences, Gerontology Research Center, University of Jyväskylä, Jyväskylä, Finland

**Keywords:** Behavior change, Aging, Quality of life, Mobility, Randomized controlled trial

## Abstract

**Background:**

We define active aging as a striving for activities as per one’s goals, capacities and opportunities.

**Aim:**

To test the 1-year counselling intervention effects on active aging.

**Methods:**

In this two-arm single-blinded randomized controlled trial, the intervention group received individually tailored counselling supporting autonomous motivation for active life (one face-to-face session, four phone calls and supportive written material, *n* = 101) and the control group written health information (*n* = 103). Participants were community-dwelling men and women aged 75 or 80 years with intermediate mobility function and without cognitive impairment. The primary outcome was active aging total score measured with the University of Jyväskylä Active Aging Scale (UJACAS, range 0–272, higher values indicate more activity) and secondary outcomes were its subscores for goals, ability, opportunity and activity (range 0–68) and a quality of life (QoL) score. Measures took place at pre-trial, mid-trial (6 months) and post-trial (12 months), except for QoL only pre and post-trial. Data were analyzed with intention-to-treat principles using GEE-models.

**Results:**

The UJACAS total score increased in the intervention group slightly more than in the control group (group by time *p*-value = 0.050, effect size 0.011, net benefit 2%), but the group effect was not statistically significant. A small effect was observed for the activity subscore (*p* = 0.007).

**Discussion:**

The individualized counselling supporting autonomous motivation for active life increased the UJACAS score slightly.

**Conclusions:**

It may be possible to promote active aging with individualized counselling, but the effect is small and it is unclear whether the change is meaningful.

## Introduction

Activity refers to doing things and relates to all essential fields of human life. Many older people state that avoiding passiveness and sustaining activeness underpins positive life experience [1, 2]. With increasing age, however, people may give up important activities e.g. due to declining functional capacity [3, 4], which may concur with lower quality of life [4].

Defining and assessing an active approach to life during aging has mostly concerned physical activity and paid or unpaid work [5]. However, adopting a broader view on activity by emphasizing participation in any meaningful activities based on individual predispositions may provide a more inclusive picture of active aging [6]. Older people have many personal goals related to diverse activities [3]. As people often act in line with their goals [3], most likely their activities are also highly diverse. We acknowledge this diversity in our recent definition of active aging as “the striving for activities relating to a person’s goals, functional capacities and opportunities” [7] which we aim to capture in our 17-item University of Jyväskylä Active Aging Scale (UJACAS). We developed the scale in a multi-phase process, and the total score represents the unidimensional latent construct of active aging. The items assess active agency in essential life areas outlined in the International Classification of Functioning, Disability and Health [8]. The four subscores assess what people want to do, what they are able to do, whether they have the opportunity to do the activity, and how much or how often they do the activity [7]. The novel aspects of UJACAS are that it captures diverse forms of activity in old age, includes generic activities that are in principle possible for all, and does not provide strict objective criteria for performing the activity. For example, for one person ‘helping others’ may mean taking part in formal and regular volunteer work, while for somebody else it may mean watering the neighbor’s plants while they are travelling.

We observed in our pilot data that the higher the active aging score was, the better were the indicators of quality of life [7] suggesting that increased activity in any essential life areas may enhance positive life experiences. Quality of life is multidimensional and refers to perceptions of one’s position in life relative to one’s goals and living environment [9]. Quality of life is increasingly included as an outcome in health interventions [10]. Due to the wide scope of the concept, many different interventions may improve quality of life among older people [10, 11].

As active aging takes diverse forms, we concluded that individualized counselling intervention could be a feasible way to promote it. Counselling is an interaction process that helps the participant to see things more clearly and possibly from a different point of view. In this study, the idea was to increase awareness of meaningful and desirable activities that are likely to yield personal benefits, to set new self-selected activity goals, promote autonomous motivation and lend support to positive changes in activity [12]. We decided to use a counselling approach that integrates two socio-cognitive theoretical models, the self-determination theory [13] and the theory of planned behavior [14]. The self-determination theory concerns motivation as our intrinsic predisposition to behave in positive ways, and it explains the motivation underlying everyday activities in older people [15–17]. The theory of planned behavior links one’s beliefs and behavior and explains how older individuals intend to behave [18–22]. A recent meta-analysis [23] suggested that integrating these two theories may provide a solid basis for planning a behavior change intervention. The integrated model has been used to elucidate the behavior of older adults [24], but earlier studies on counselling interventions based on the integrated model do not exist.

The aim of this 12-month randomized controlled trial was to investigate the effects of the individualized counselling intervention on the UJACAS total score (primary outcome). In addition, we examined the effects of the intervention on the four UJACAS sub-scores and quality of life (secondary outcomes). The hypothesis was that the UJACAS total score will increase 10% in intervention group and no change or slight decrease will be observed in the control group. For future hypothesis building, we conducted preplanned sub-group analyses for the UJACAS total score.

## Methods

The study protocol has been published earlier [12]. The study design was a single-blinded randomized controlled trial with two research arms. The parallel groups are the “Counselling group”, serving as the intervention group, and a “Health information group”, serving as the control group. Trained research assistants collecting data at all time-points were unaware of the group allocation.

### Participants

We recruited participants from the AGNES cohort study [25, 26], which is an observational study on active aging of people aged 75, 80, or 85 years living independently in the municipality of Jyväskylä, Finland. Participants for the cohort study were recruited based on random samples drawn from the Population Information System administered by the Population Register Center based on their place of residence and year of birth. All who consented and were able to communicate with researchers were included in the cohort study. Inclusion criteria for the current RCT were age 75 or 80 years, willingness to participate, a baseline score between 52.3 and 90.0 on the University of Alabama at Birmingham Life-Space Assessment (LSA) [27, 28], and a score of 25 or higher on the cognitive function test Mini Mental State Examination (MMSE) [29]. Life-space mobility describes the spatial area where a person moves on a regular basis, the frequency of moving and the assistance needed [26]. Consequently, it describes a person’s prospects to take part in different life-situations. People with life-space mobility composite score in the recruitment range represent the “middle group” in the population, excluding those with mobility restrictions and those with most extensive mobility behavior. We selected the MMSE score 25 or higher as an inclusion criterion because counselling is a cognitive intervention and requires the participant to process the topics discussed. We excluded people who took part in other ongoing intervention studies. Recruitment started in October 2017 and continued until end of August in 2018. Measurements were completed in August 2019.

### Randomization

We used stratified randomization for age and sex with a 1:1 allocation to ensure a balance of participant characteristics in each group. The randomization sequence was created using Stata 15.0 statistical software (StataCorp, College Station, TX) by the study statistician, who immediately after generation of the random allocation sequence sealed them in envelopes. For each person, the counsellor opened the envelope after completion of all baseline data collection.

### Measurements

Participants were assessed pre-trial, mid-trial at 6 months, and post-trial at 12 months by trained research assistants. All assessments were conducted face-to-face in the participants’ homes by trained interviewers using computer assisted personal interviewing. In addition, prior to randomization the counsellor conducted a brief interview over the phone for profiling purposes.

### Intervention

The active aging counselling intervention has been described earlier [12]. Briefly, the counselling group participated in a 90-min, face-to-face, individual counselling session on the premises of the University of Jyväskylä following a semi-structured protocol of questions. After the initial face-to-face session, participants received four phone counselling sessions, at 1, 3, 6 and 9 months after the face-to-face counselling session to provide them with additional support, feedback and encouragement. Participants received four printed newsletters listing activities organized for older people in our town, and the success stories of people who are living an active life. During their face-to-face counselling session, participants received an information booklet on active aging from the counsellor. The booklet is 64 pages long and provides information about goal setting for active life, and activities and behaviors that promote active aging.

The central elements of the counselling intervention were to support the autonomous motivation for and foresee the benefits of increased or new activities according to the goals set by the participant. We also provided information about different local activities for older people. The 17 activities composing the UJACAS were used as the starting point for the goal setting discussions. As a result, the targets were several different active behaviors that differed between individuals. Depending of the preferences of the participant, we encouraged any self-selected activities that take place outside the home or that involve social interaction, such as attending events, going for walks with a friend or joining a club. Our unpublished pilot data analyses suggested that such activities have largest ‘spill-over’ effects on other activities and quality of life. We followed the guidelines of the Medical Research Council (MRC) framework for complex interventions [30] and implementation research [31–33].

To support autonomous motivation for self-selected activities, individuals set their goals, planned their actions and monitored their progress in change process. The individualization of the counselling sessions rested on pre-existing data on health, social contacts, mood, loneliness and preferred activities and goals.

The health information group received printed general health information material similar to what is used in health care services for older people. We posted the printed materials (brochures, booklets etc.) to the health information group participants during months 1, 3, 6, and 9. The themes were exercise, nutrition, cardiovascular diseases, and type II diabetes, respectively.

### Main outcome

The main outcome measure is the total score of the University of Jyväskylä Active Aging Scale (UJACAS) [7]. The UJACAS consists of 17 items: practicing memory, using a computer, advancing matters in one’s own life, exercising, enjoying the outdoors, taking care of one’s appearance, crafting or DIY, making the home cozy and pleasant, helping others, maintaining friendships, getting to know new people, balancing personal finances, making one’s days interesting, practicing artistic hobbies, participating in events, advancing societal/communal matters, and doing things according to one’s world view. For each item, participants were asked to evaluate on a Likert scale their striving to accomplish the activity, their ability and opportunity to perform the activity and their amount or frequency of doing the activity during the 4 weeks immediately prior to the measurement. Response options ranged from zero (not at all/very low) to four (very much/very high) with verbalization of rating depending on the wording of the question. Subscores (range 0–68) for the striving, ability, opportunities and activity were then calculated and the composite score (range 0–272) were calculated with higher scores reflecting greater striving, better ability or opportunities, and higher activity. Similarly, a higher composite score (range 0–272) indicates a higher level of active aging. We have previously shown that the scale has good psychometric properties, test–retest reliability and that it assesses a unidimensional latent construct of active aging [7].

### Secondary outcomes

The secondary outcomes include the active aging subscores and quality of life score.

Quality of life was assessed pre- and post-trial with a short version of the Quality of Life Questionnaire for Older People (OPQOL-brief). The scale includes 13 items related to a person’s satisfaction with life overall, health, participation, social relationships and financial situation. Answers are given on a scale from one (strongly disagree) to five (strongly agree). The sum score ranges from 13 to 65, with higher scores indicating higher quality of life [34].

### Additional baseline measures

Age and sex were obtained as part of the sampling data drawn from the Population Register Centre in the context of recruitment. Education was assessed by the number of completed years of education [35]. Perceived financial situation was assessed on a scale ranging from one (very good) to five (very poor). Physical health was assessed based on self-reported physician-diagnosed diseases. A co-morbidity index was calculated from a checklist of diseases prompted by ten categories of chronic diseases and an open-ended question about any other physician-diagnosed chronic conditions [36]. Lower-extremity physical performance was assessed by the Short Physical Performance Battery (SPPB) [37]. It comprises tests on standing balance, walking speed over a 3-m distance, and chair-stands. Established cut-off points were used to score each task from zero to four points, higher scores indicating better performance. Participants unable to perform a test were assigned the score zero. A sum score was calculated (range 0–12) when at least two tests were completed. Cognitive functioning was tested with MMSE [29] with higher scores indicating better results.

### Statistical analyses

Power calculations were conducted for the primary outcome, the UJACAS total score assessed at 12 months. A total of 168 participants were needed for a 90% probability to detect a treatment difference at a two-sided 0.05 significance level, if the true difference in the main outcome between the intervention and the control group is 10%. We assumed that some of the participants are vulnerable, so we decided to recruit 200 participants to allow for the potential attrition rate of 20% during the 12-month intervention. The estimate of 10% difference between the groups was based on approximations from two earlier intervention studies [37, 38].

Baseline characteristics were summarized using means and standard deviation, or percentages. Intervention adherence was calculated based on the proportion attending the scheduled face-to-face and phone counselling sessions. We analyzed the effect of the intervention on the primary outcome, the active aging total score, with intention-to-treat approach according to randomized groups. To compare intervention effects linear Generalized Estimation Equations (GEE) for repeated measures were computed using unstructured correlations, where group × time interaction indicates if change differs between the groups. This method adjusts for potential baseline differences in the outcomes. GEE modeling takes into account all available data when estimating intervention effects. Effect size was calculated from per protocol analyses and describes the net difference in changes between groups. The group difference represents the difference in the level of the active aging score over the entire follow-up. The secondary endpoints were analyzed similarly as the primary outcome.

The trial is registered at ISRCTN—ISRCTN16172390: promoting well-being through active aging.

## Results

**Fig. 1:**
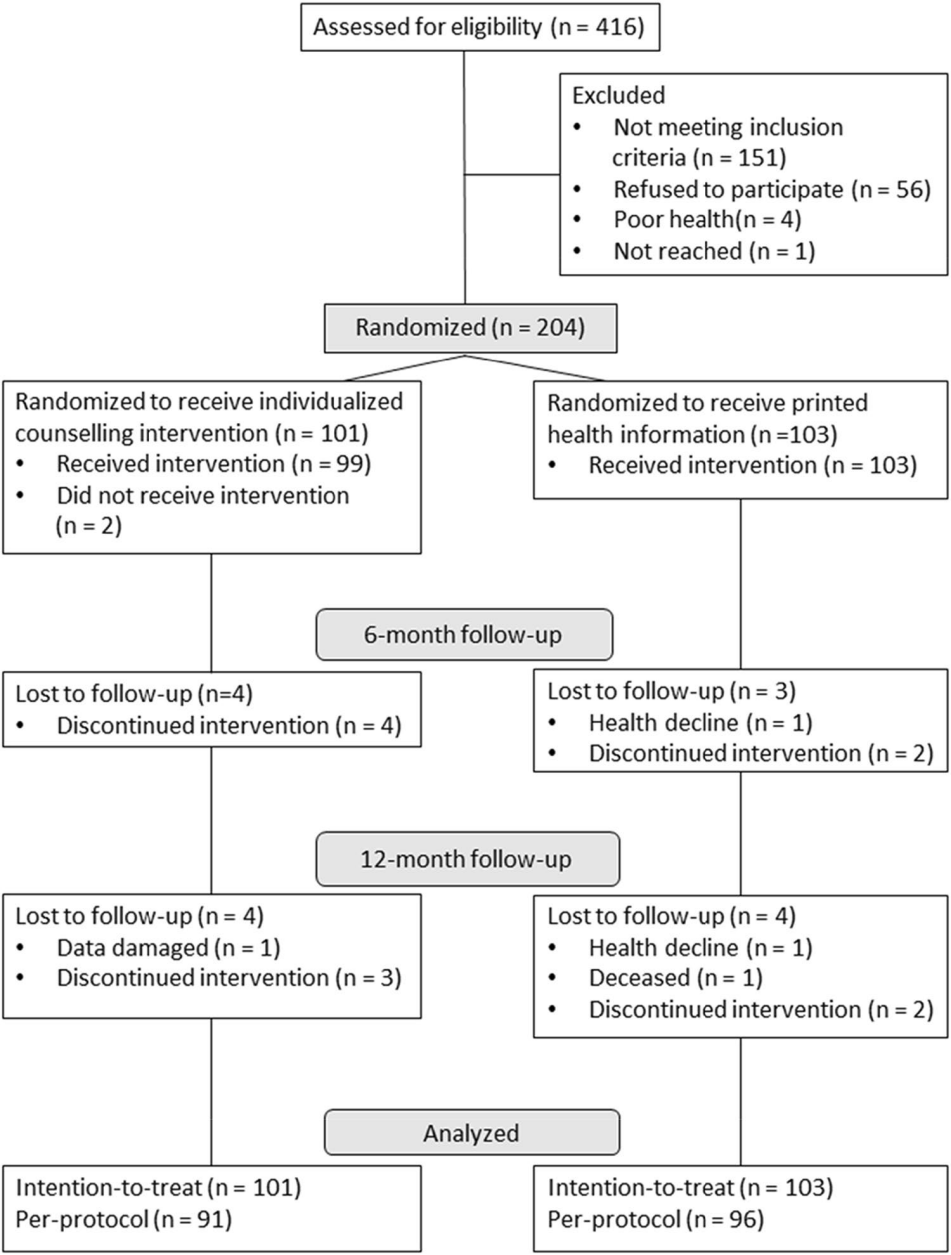
The flow chart of the study

From October 2017 until end of August in 2018, we screened 416 participants of the AGNES-cohort study for the current RCT. Of them, 204 were eligible and were randomized either to the active aging counselling group (*N* = 101) or to the health information group (*N* = 103) (Fig. 1). Baseline characteristics between the two groups were comparable (Table 1). All participants were aged 75 or 80 years and approximately a quarter of both groups were 80 years of age and approximately 60% were women. The sole statistically significant difference observed was for SPPB score with health information group receiving slightly higher results at the baseline.

**Table 1:** The baseline characteristics of the participants according to randomized groups

	Counselling (*n* = 101)	Health information (*n* = 103)
Age, 80-year-olds, *n* (%)	26 (25.7)	26 (25.2)
Women, *n* (%)	61 (60.4)	63 (61.2)
Tertiary education, *n* (%)	41 (41.0)	53 (51.5)
Education, mean (SD), years	11.4 (3.9)	12.0 (3.7)
Perceived financial situation, very good/good, *n* (%)	60 (60)	66 (66)
Life-space mobility, mean (SD), score	75.5 (9.2)	74.7 (9.3)
MMSE, mean (SD), score	27.8 (1.5)	28.2 (1.2)*
CES-D, mean (SD), score	8.12 (7.1)	7.0 (6.1)
SPPB, mean (SD), score	10.6 (1.6)	11.0 (1.2)*

*CES-D* Center for Epidemiologic Studies Depression Scale, *SPPB* Short Physical Performance Battery, *MMSE* mini-mental state examination, *p =  < 0.05

### Intervention adherence

Of the 101 participants randomized into the counselling group, two did not receive any part of the intervention while seven participants cancelled their participation later on during the intervention. Altogether, 8.9% discontinued in the intervention. In the control group, seven participants (6.8%) discontinued.

### Adverse events

The counselling and health information groups did not differ in incidence of adverse events related to health and social relationships during the intervention study. In the counselling group, 20% of the participants got a new disease, in 20% existing disease got worse, 8% had an accident, 17% had a medical operation, and 44% some other health problem. Proportions in the health information group were 18%, 22%, 5%, 11% and 39% respectively. Death of a close person occurred for 11% in the counselling and 19% in the health information group. In the counselling group, 26% worried about health or life situation of a close person and 6% reported of narrowed friendship network. Respective proportions for the health information group were 28 and 2%.

One person reported negative effects directly related to the study, which was feeling exhausted during the 6-month interview.

### Active aging scores

According to the principles of the ITT-analyses all participants randomized were included in the GEE models testing the intervention effects. Table 2 shows that the counselling intervention increased the active aging total score compared to the health information (group by time *p*-value = 0.050). The net difference in change was 2% for the benefit of the intervention.

**Table 2:** Means and standard deviations (SD) for the active ageing total score (main outcome) and active ageing subscores at the baseline, 6 months and 12 months in the randomized groups

Outcome	Baseline Mean (SD)	6 Months Mean (SD)	12 Months Mean (SD)	Group *p*-value	Time *p*-value	Group by time *p*-value	Interaction effect size
Active ageing, total score				0.121	0.153	0.050	0.011
Counselling group	204.1 (26.3)	200.6 (25.7)	207.2 (24.6)				
Health information group	207.9 (25.3)	203.0 (24.6)	206.6 (24.1)				
Will to act, subscore				0.483	0.614	0.369	0.005
Counselling group	45.7 (9.3)	45.1 (8.6)	46.8 (8.7)				
Health information group	46.2 (8.7)	46.2 (8.6)	46.8 (8.2)				
Ability to act, subscore				0.284	0.023	0.162	0.007
Counselling group	62.1 (6.0)	60.7 (6.8)	62.0 (6.4)				
Health information group	62.4 (5.4)	60.9 (6.3)	61.3 (6.5)				
Opportunity to act, subscore				0.231	0.065	0.279	0.003
Counselling group	54.3 (9.2)	52.3 (9.5)	54.1 (8.8)				
Health information group	55.5 (7.9)	53.0 (8.5)	54.5 (7.8)				
Amount of activity, subscore				0.045	0.793	0.007	0.019
Counselling group	42.1 (8.4)	42.4 (7.5)	44.4 (7.1)				
Health information group	43.9 (8.6)	43.0 (7.6)	44.0 (7.6)				

Generalized estimation equations with *p*-values for the main effects of group and time, and group by time interaction shown for each outcome. Effect size is calculated for per protocol participants

For the active aging subscores, a significant group by time interaction was observed for the amount of activity (*p* = 0.007) accompanied by a significant group difference (*p* = 0.045) with intervention group getting higher values (Table 2). For the other subscores, statistically significant effects were not found.

The mean quality of life score at the baseline was 56.2 (SD 4.8) and the intervention had no effect on quality of life, the groups did not differ, and statistically significant change over time was not observed.

### Preplanned sub-group analyses

The preplanned subgroup analyses for the main outcome were conducted for age, sex, socioeconomic position, MMSE and SPPB (Table 3). The intervention increased active aging total score among those with self-rated poor or at most satisfactory financial situation (group by time *p*-value 0.033) and those with high SPPB score (group by time *p*-value 0.038).

**Table 3:** Means and standard deviations (SD) for the active ageing total score for counselling group vs. health information group according to subgroups at the baseline, 6 months and 12 months in the randomized groups

Subgroup	*N*	Baseline Mean (SD)	6 Months Mean (SD)	12 Months Mean (SD)	Group *p*-value	Time *p*-value	Group by time *p*-value
Women					0.065	0.865	0.123
Counselling group	61	203.8 (26.1)	176.7 (68.6)	206.8 (25.0)			
Health information group	63	210.6 (24.3)	193.0 (55.2)	209.1 (22.8)			
Men					0.081	0.598	0.279
Counselling group	40	204.6 (26.9)	200.9 (23.6)	207.8 (24.4)			
Health information group	40	203.7 (26.4)	192.6 (40.9)	202.7 (25.8)			
75 year-olds					0.309	0.068	0.093
Counselling group	75	205.4 (24.8)	193.1 (47.2)	206.3 (24.0)			
Health information group	77	208.0 (26.0)	193.0 (46.3)	205.4 (25.2)			
80 year-olds					0.421	0.811	0.805
Counselling group	26	200.5 (30.7)	166.8 (74.8)	210.3 (27.0)			
Health information group	26	207.5 (23.5)	192.1 (60.4)	209.9 (20.9)			
Financial situation very good/good					0.863	0.299	0.460
Counselling group	60	210.8 (25.0)	101.1 (38.8)	210.9 (23.7)			
Health information group	63	208.1 (24.8)	192.0 (54.0)	208.0(14.5)			
Financial situation satisfactory/poor					0.019	0.387	0.033
Counselling group	40	195.3 (24.9)	165.4 (73.3)	202.3 (24.5)			
Health information group	35	207.4 (26.5)	194.4 (41.6)	204.1 (23.6)			
MMSE 30–28					0.194	0.140	0.155
Counselling group	61	207.5 (26.3)	188.4 (56.3)	201.1 (24.9)			
Health information group	71	211.5 (25.4)	199.2 (42.5)	209.1 (25.3)			
MMSE 27–25					0.603	0.711	0.214
Counselling group	40	199.0 (25.8)	183.0 (57.2)	202.8 (23.9)			
Health information group	32	200.0 (23.5)	178.6 (61.8)	200.5 (20.1)			
SPPB 12–11					0.282	0.065	0.038
Counselling group	64	211.5 (23.8)	199.3 (44.0)	213.0 (23.2)			
Health information group	73	212.5 (24.6)	196.8 (27.9)	209.2 (25.5)			
SPPB 10–5					0.313	0.919	0.464
Counselling group	37	191.3 (25.9)	163.8 (68.1)	197.1 (24.0)			
Health information group	30	196.7 (23.5)	183.1 (54.3)	200.7 (19.5)			

Generalized estimation equations with *p*-values for the main effects of group and time, and group by time interaction are shown

## Discussion

The intervention increased active aging total score statistically significantly, but the effect size and net benefits were small. The results suggest that it may be possible to promote active aging with individualized counselling targeting autonomous motivation for self-selected activities, however, it is unclear whether the observed small changes are meaningful. This is the first study to target a wide range of self-selected activities with individualized counselling supporting autonomous motivation.

We planned the study based on the results of our earlier observational studies and randomized controlled trials. We reported previously that having more personal goals correlates with greater life-space mobility, which exemplifies a person’s exposure to out-of-home goings-on [38]. Any activity outside the home will increase physical activity and social interaction and potentially help prevent social isolation and physical inactivity, see e.g. [39, 40]. Our earlier physical activity counselling intervention increased physical activity and maintained better mobility [41, 42]. For the current study, we extended the scope to any self-selected activity that takes place outside home or that is done with other people and incorporated new knowledge on behavior change techniques into the intervention [12]. Motivation is a psychological concept defined as ‘a driving force for the goal-directed behavior’ [13]. Consequently, in the current study we aimed to promote autonomous motivation for self-selected activities [12]. Autonomous motivation relates to personal importance rather than extrinsic control [13].

The results showed that the secondary outcome ‘will to act’ did not change, but ‘activity’ increased. Activity was indicated as the sum of the accumulated participation in the 17 active behaviors forming the UJACAS activity subscore. Our expectation was that the counselling intervention component would mainly increase the subscore ‘will to act’ and that the booklet and newsletters would contribute to the changes of ‘ability to act’ and ‘opportunity to act’ respectively. As a result of the interaction of those elevated subscores the activity subscore would increase and the total score would increase. The results showed that even though the change of the secondary outcome ‘will to act’ was insignificant, the change of the ‘activity’ score was significant. The results may imply that even a small change in motivation may produce significant behavioral changes. However, this topic needs further research. The subscore ‘opportunity to act’ did not change. The ‘ability to act’ scores did not change either. The responses concerning ability to act clustered toward the higher values, suggesting, first, that there was not much room for change, and second, that the participants were well able to do the activities and thus inability to act did not prevent them from doing what they want to do.

The preplanned subgroup analyses suggested that people with good lower extremity performance (SPPB score 11–12) or lower self-perceived financial situation benefitted more. However, all the observed effects were modest. These results should not be interpreted as intervention effects but rather they lay foundations for planning future studies.

There may be some explanations for the lower than expected intervention effect in the active aging score. It is possible that our inclusion criteria was not optimal. Our goal was to target the ‘middle group’ in terms of activity in order to find a group of people who had room for improvement and in the same time also resources to increase their activity. However, using the life-space mobility score between 52.3 and 90.0 on the University of Alabama at Birmingham Life-Space Assessment (LSA; [27, 28]) may have resulted in including people with highly active approach to life to start with. The subscale analyses suggested that the change in the total score resulted from the changes in the activity subscale. It is possible that the subscales relating to ability and opportunity to do different activities may not respond to a counselling intervention, but instead may require environmental modifications, e.g. changes in the physical living environment or social context. In the future in similar interventions, it might be better to choose the UJACAS activity subscore as the main outcome and the other subscores as secondary outcomes, because they may describe factors underlying activity. We recruited participants from a population-based probability sample, and the participants were not self-referred. This reduces bias towards higher intervention effectiveness often observed in studies among convenience samples. It is also possible that our active aging scale in terms of items and categorizations did not capture the changes caused by the intervention. As the items did not have strict external criteria for specific activities, it is possible that the activities changed to more demanding forms, but the scale did not detect that. It is also possible that the generic scale with 17 different activities made it difficult to capture changes, because each participant selected one to three activities that they aimed to increase. The anecdotal information from the participant feedback forms suggests that many participants perceived that the counselling promoted their active agency, some participants did not perceive a need for significant changes in their behavior, while some had so problematic issues in their lives that could not be solved by the current intervention. Finally, we need to admit that behavior change is difficult. A meta-analysis on the effectiveness of face-to-face interventions for promoting physical activity for at least at 12 months concluded that the effectiveness of these interventions was not supported by high quality studies [43].

The strengths of the current study include the randomized controlled design and incorporating into the intervention the up-to-date behavioral change techniques, such as setting goals and planning, getting feedback and monitoring, social support and monitoring consequences. Our study attrition was low. The main outcome was the novel active aging score not previously studied. The intervention lasted for one year. Consequently, the baseline and the final assessment took place in the same season. Even though the intervention effect was low, it is still notable that in our rather homogenous sample, there was a significant change considering that it is very difficult to change behaviors.

The weaknesses include that we do not yet know how big a change in the active aging score is meaningful. However, the responses are self-assessments, and any change in self-report may be considered meaningful, because it is based on the participants’ immediate experience about their life. We do not know whether the participants excluded from the study based on their too low or high life-space mobility score would have benefitted more from the intervention. The preplanned subgroup analyses suggest that the active aging score increased more among those with intact lower extremity performance and lower self-rated financial situation. Excluding people with lower MMSE scores might not have been necessary, because the subgroup analyses showed that the results did not differ between those in the higher or lower MMSE score.

An individualized counselling intervention based on self-determination theory and theory of planned behavior, and consisting of one face-to-face session, an information booklet, four phone counselling sessions and four printed newsletters featuring the available activities organized in our town increased the active aging score modestly compared to the health information intervention. These findings suggest that it is possible to promote active aging by focusing on the individuals’ goals. Future studies on active aging should include samples with higher variability and incorporate environmental interventions to influence opportunities in addition to individual-based intervention.

## Data Availability

After completion of the study, data will be stored at the Finnish Social Science Data Archive without potential identifiers (open access). Until then, pseudonymized datasets are available to external collaborators upon agreement on the terms of data use and publication of results. To request the data please contact Professor Taina Rantanen (taina.rantanen@jyu.fi).
